# Impacts of Common Milkweed (*Asclepias syriaca*) Leaf Age on Larval Monarch (*Danaus plexippus*) Survival, Growth, Development, and Feeding Behavior

**DOI:** 10.3390/insects17020215

**Published:** 2026-02-19

**Authors:** Kelsey E. Fisher, Caleb B. Bryan, Cody Acevedo, Kevin E. Anderson, Kira M. Goldman, Karena Kulakowski, Samantha N. Shimota, Steven P. Bradbury

**Affiliations:** 1 Department of Entomology, Connecticut Agricultural Experiment Station, New Haven, CT 06511, USA; 2 Department of Natural Resource Ecology and Management, Iowa State University, Ames, IA 50011, USA; 3 Department of Plant Pathology, Entomology and Microbiology, Iowa State University, Ames, IA 50011, USA

**Keywords:** host plant quality, phenology, habitat management, nutritional ecology, insect-plant interactions

## Abstract

Milkweed (*Asclepias* spp.) is the sole host plant for monarch butterfly (*Danaus plexippus*) larvae, yet its quality varies within and among individual plants. This study examined how leaf position, used as a proxy for leaf age, on *Asclepias syriaca* influences monarch larval performance and feeding behavior. We found that younger leaves from the tops of milkweed stems had higher nitrogen levels and were easier to consume than older, lower leaves. Larvae that fed on top leaves grew larger, developed faster, and exhibited consistently higher survival rates, although the magnitude of these effects was modest. In choice bioassays, both neonates and third instars showed preference for younger leaf tissue, suggesting larvae can differentiate leaf types. These findings indicate that within-plant variation in milkweed quality can affect monarch fitness. Because milkweed quality declines as plants mature, periodic disturbance (e.g., mowing or burning) that promotes regrowth may improve leaf quality and larval performance. However, such practices involve trade-offs, including potential impacts on other species and potential disruption or diapause cues and migration timing. Selective or rotational mowing may offer a more sustainable approach by balancing regrowth benefits with preservation of natural phenological cues.

## Simple Summary

Monarch butterfly (*Danaus plexippus*) larvae grew larger and had improved survival when feeding on younger leaves located at the top of *Asclepias syriaca* (common milkweed) plants. These leaves contained higher nitrogen levels and were more tender than older leaves, making them easier to consume. Behavioral assays confirmed that larvae preferentially select younger foliage. While these findings suggest that younger milkweed growth can improve larval performance, the benefits were modest, and management practices such as mowing involve trade-offs, including potential impacts on other species, diapause cues, and migration timing. Selective or rotational mowing may help balance these considerations while maintaining habitat quality.

## 1. Introduction

Monarch butterflies (*Danaus plexippus*) are among North America’s most iconic butterfly species, widely recognized for their striking orange and black coloration and their remarkable multi-generational migration. Monarch larvae are obligate herbivores of milkweed (*Asclepias* spp.), making host plant availability and quality critical factors to their survival and development. Conservation interest in monarchs remains high, as reflected in recent U.S. Fish and Wildlife Service (USFWS) assessments and proposed listing decisions [1,2]. Although milkweed abundance has received considerable attention in terms of larval survival and development, comparatively less research focus has been placed on the spatial distribution and quality of milkweed within habitats and how these factors influence monarch fitness [3]. Our study advances understanding of how fine-scale variation in milkweed quality influences larval performance and behavior, which may inform habitat management practices.

The spatial arrangement of milkweed in the landscape plays a critical role in shaping monarch oviposition patterns and larval development outcomes. Milkweed tends to be unevenly distributed across the landscape, often occurring in clumps or as isolated ramets [4,5]. Importantly, monarch females do not utilize milkweed uniformly for oviposition. Observations from studies of monarch egg and larval distributions show that anywhere from three to ten eggs may be laid within a milkweed cluster or on an isolated ramet at any given time [5,6]. Simulation modeling suggests that higher egg densities are likely to occur in landscapes with smaller, more fragmented habitat patches compared to large, contiguous habitat areas [3,4,7,8]. As monarch larvae generally consume the biomass equivalent of two or three ramets during development [9], isolated ramets may be insufficient to support complete development, even though they tend to accumulate more eggs. These spatial constraints may influence larval movement, survival, and overall habitat suitability.

In addition to spatial distribution, milkweed quality may influence monarch oviposition and larval performance. The quality of milkweed can be characterized by nutritional content, leaf toughness, and defensive traits. These characteristics vary among milkweed species [10]. Typically, high-quality milkweed contains high nitrogen concentrations, supple leaves, and moderate defenses, including intermediate cardenolide concentrations, latex production, and trichome density [10,11,12,13,14,15]. Milkweed species with these traits are often preferred in oviposition studies and tend to support greater growth, development, and survival [16,17,18,19].

Plants also exhibit substantial within-plant variation in leaf traits, which differ based on developmental stage and phenology [20]. Although young milkweed vegetation is typically defended by higher cardenolide concentrations and greater latex flow [13,21], young leaves are generally smaller, more supple, and more nutrient-rich than older leaves [20,22,23,24,25]. Monarch females appear to favor these younger, more succulent leaves for oviposition [26,27,28,29,30,31,32], and larvae are most often observed on the upper portions of milkweed ramets where new growth is concentrated [9,33]. Fine-scale differences in leaf quality may influence larval lepidopteran feeding behavior, growth, and survival [22,24,34,35], yet few studies have explored how monarchs respond to such detailed variation in host plant quality, as noted by Haan and Landis [36].

One particularly intriguing behavior that may be influenced by within-plant variation in milkweed quality is natal ramet abandonment [9]. This appears to be an innate behavior, observed in both field and greenhouse settings [9,26,37,38,39,40,41], in which a larva abandons its milkweed ramet before completing development despite the presence of remaining edible plant biomass. In previous studies, under controlled conditions, all larvae exhibited this behavior, leaving their natal plant regardless of resource availability [9,41]. Most frequently, this abandonment occurred after approximately six days, during the fourth instar, with only 25–50% of the plant consumed [9,41]. Of note, the 25–50% of the plant that was consumed was generally on the top of the milkweed plant where new growth is concentrated [9]. This behavior occurred in the absence of competition, predation, or forage limitation, suggesting an intrinsic behavioral response. Larvae may leave their natal plant due to declining plant nutritional quality, increased induced defenses, or structural changes such as reduced leaf cover [9,41]. Notably, this movement appears to be random rather than directed toward nearby host plants. As a result, natal ramet abandonment could negatively impact monarch fitness in fragmented or isolated habitats, where larvae are less likely to encounter another suitable milkweed ramet after leaving their natal plant. Because this behavior occurs independent of external stressors, it may be a consistent feature of larval development that should be accounted for in habitat design.

Here, we tested the hypothesis that plant quality declines as milkweed matures and that monarch larvae respond both physiologically and behaviorally to this change. Throughout this study, we focus on within-plant variation in host quality and larval-stage responses. Specifically, we hypothesized that: (1) upper leaves on a milkweed ramet are higher in quality than older, lower leaves on the same ramet; (2) leaves from the top of a milkweed ramet support greater survival, growth, and development than leaves from the bottom of the plant; and (3) monarch larvae preferentially feed on upper leaves when given a choice. To address these hypotheses, we investigated how leaf position on *Asclepias syriaca* affects monarch larval performance and feeding behavior. We quantified differences between upper and lower leaves, tracked growth and survival across multiple trials, and conducted no-choice and choice feeding assays with neonates and third instars. By integrating plant trait measurements with developmental and behavioral assays, our study provides new insights into how fine-scale variation in host plant quality shapes monarch fitness outcomes. Importantly, this work also offers indirect evidence for the potential physiological and behavioral drivers of natal plant abandonment, a behavior that may be triggered by localized declines in host plant quality.

## 2. Materials and Methods

### 2.1. Insects

Monarch eggs were acquired from colonies maintained by the United States Department of Agriculture (USDA), Agriculture Research Service (ARS), Corn Insects and Crop Genetics Research Unit (CICGRU) in Ames, IA. Maintenance of these colonies follows standard management practices designed to minimize disease prevalence, including regular monitoring and mitigation of *Ophryocystis elektroscirrha* (OE) infestations. To minimize the potential impacts of inbreeding, eggs used in each trial were sourced from colonies established the year prior to experimentation (e.g., 2017 colony for 2018 experiments, 2018 colony for 2019 experiments). Eggs were laid on greenhouse-grown *Asclepias curassavica* and incubated in a growth chamber under controlled conditions: 16:8 light:dark photoperiod, 23 °C, and approximately 50% relative humidity. Experiments were initiated with neonates collected within five hours of hatching. For experiments initiated with third instars, larvae were reared on greenhouse-grown *Asclepia syriaca* under laboratory conditions prior to the start of the trial.

### 2.2. Plants

Field-grown *Asclepias syriaca* was sourced from community gardens, habitat restoration sites on farm properties and in city parks, and roadside rights-of-way. For use in experiments, leaves were collected from the top and bottom thirds of vegetative-stage milkweed plants ranging from 10 to 35 cm in height. Leaf position was used as a proxy for leaf age, as new vegetative growth emerges from the top of the plant. These milkweed sources were not intentionally exposed to herbicides or insecticides. Although incidental pesticide contamination of field-grown milkweed is possible [42], leaves used in experiments were randomly assigned to larvae. Therefore, any low-level background residues would be expected to be evenly distributed across treatments and unlikely to bias comparisons. Leaves were stored in separate plastic bags with a water-moistened paper towel and kept refrigerated at 4 °C for up to three days prior to use.

### 2.3. Milkweed Quality

The lengths and widths of 144 leaves from the top portion and 144 leaves from the bottom portions of 72 milkweed plants (two top leaves and two bottom leaves per plant) were measured using a ruler. To measure leaf toughness, the force required to puncture each leaf was assessed using a penetrometer (Nidec-Shimpo, Model FGE-20XY, Kyoto, Japan) equipped with a puncture test attachment (Model 49017-10), similar to methods described by Agrawal and Fishbein [10]. After these measurements were recorded, leaves were dried in a drying oven for one week. Dried leaf material from 10 top leaves and 10 bottom leaves (paired from 10 plants) was individually ground into fine powder using a mortar and pestle. Leaf powder samples were weighed (0.10049–0.10832 g), sealed in tin capsules, and submitted to the Analytical Chemistry Department at the Connecticut Agricultural Experiment Station in New Haven, CT, for nitrogen analysis. Total nitrogen content was measured using a LECO FP828 Nitrogen Determinator (LECO Corporation, Saint Joseph, MI, USA). Cellulose and a certified reference material (ethylenediaminetetraacetic acid, EDTA, Sigma-Aldrich, St. Louis, MO, USA) were included as reference standards for quality assurance purposes.

### 2.4. Survival, Growth, and Development

Across 2023 and 2024, four trials were conducted to quantify the impact of milkweed leaf position (top or bottom) on monarch survival, growth, and development, following methods similar to Fisher et al. [43]. For each trial, neonates within 5 h of hatching were placed individually into rearing cups containing either top or bottom leaves from a milkweed stem (60 larvae per treatment group). Leaves were replaced daily, and larvae were monitored for instar progression. If a larva was found dead, the cup was removed from the trial. If a larva was missing, the cup was monitored for two additional days without replacing leaf material. Larvae that remained missing for three consecutive days were recorded as dead. On the sixth day of development and again 24 h after pupation, surviving individuals were weighed using a precision balance (Mettler Toledo NewClassic MF, Model MS304S; Mettler Toledo, Columbus, OH, USA) to assess growth and final mass. Butterflies were sexed and weighed 24 h after eclosion for three of the four trials.

### 2.5. Feeding Behavior Assays

To determine feeding preference, 24 h no-choice and choice experiments were conducted with neonates and third instars in 2017, 2018, and 2023, following methods similar to Fisher et al. [44]. Four leaf disks were excised using a 1.75 cm-diameter brass cork-borer (Humboldt Manufacturing Company, Elgin, IL, USA) and were photographed using an iPhone 12 Pro (Apple Inc., Cupertino, CA, USA). Disks were placed in 9 cm Petri dishes (Fisher Scientific, Waltham, MA, USA) on water-moistened 9 cm filter paper (Whatman, Buckinghamshire, UK).

In no-choice assays, all four leaf disks originated from the same position on a single milkweed stem (either top or bottom). In choice assays, each Petri dish contained two disks from top leaves and two from bottom leaves of the same plant, placed in diagonally opposite quadrants. To reduce positional bias, the orientation of leaf disks within each dish was randomized. A randomly selected neonate or third instar was weighed and placed in the center of the dish. After 24 h, larvae were removed and reweighed. Remaining leaf disks were photographed, dried in a drying oven for one week, and weighed. Biomass consumed was estimated by calculating the area of the leaf removed using ImageJ version 1.54p [45] and multiplying by the dry mass of each disk.

Due to occasional mortality during the 24 h period, final sample sizes varied. For no-choice assays, there were 80 replicates across four trials for neonates and 94 replicates across four trials for third instars fed top leaf disks, and 79 replicates across four trials for neonates and 87 replicates across four trials for third instars fed bottom leaf disks. For choice assays, there were 79 replicates across six trials for neonates and 98 replicates across five trials for third instars, each provided with a choice between top and bottom leaf disks.

### 2.6. Statistical Analyses

All statistical analyses were conducted in RStudio version 4.3.1 [46] using the package emmeans [47]. The primary independent variable was the position on the milkweed stem from which a leaf originated (top or bottom). Milkweed quality characteristics, including leaf length, width, toughness, and nitrogen content, were analyzed with generalized linear models (GLMs) that accounted for site and date of milkweed collection. Monarch survival proportions at day six of larval development and at pupation were analyzed using chi-square tests for proportions (chisq.test). Larval mass on day six, pupal mass, female and male adult masses, and days to pupation were analyzed with GLMs that included trial replicate and year as covariates. Biomass consumed in no-choice and choice feeding assays for both neonates and third instars, as well as larval mass change in no-choice assays, were analyzed using GLMs accounting for trial replicate. Although year and trial often had significant effects, these variables were removed from the final models to increase statistical power, as these variables did not interact with the primary treatment effect.

## 3. Results

### 3.1. Milkweed Quality

Leaf characteristics varied significantly between milkweed leaves from the top and bottom portions of the plant (Table 1). Leaves from the top were approximately 20% shorter and 35% more narrow in length and width compared to those from the bottom of the plant. Top leaves required ~18% less force to puncture than bottom leaves, indicating they were structurally softer. Additionally, top leaves contained significantly more nitrogen than bottom leaves.

### 3.2. Survival, Growth, and Development

There was no statistically significant difference in the proportion of larvae surviving until day six (χ^2^ = 0.0096257; df = 1; *p* = 0.9218) or until pupation (χ^2^ = 1.3139; df = 1; *p* = 0.2517) between individuals fed leaves from the top or bottom of milkweed plants. However, survival rates were numerically higher for larvae fed top leaves (75.4% on day six; 60.8% at pupation) compared to those fed bottom leaves (74.2% on day six; 48.8% at pupation).

**Figure 1:**
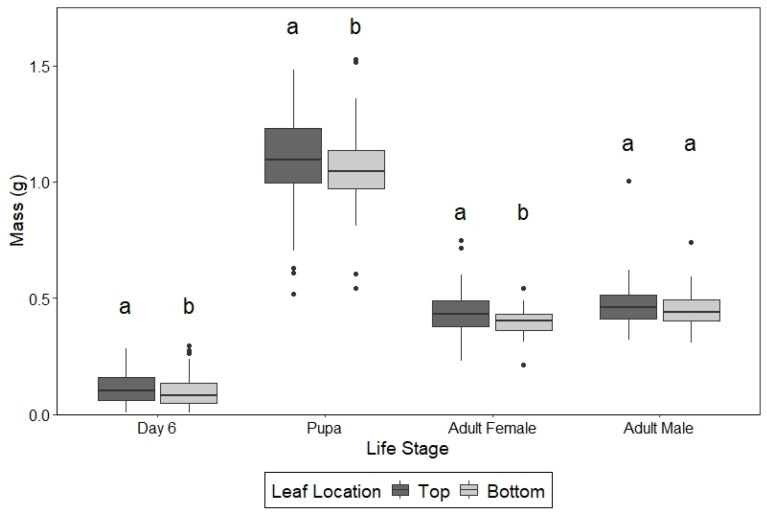
Boxplots of the mass on day 6 of larval development, 24 h after pupation, and 24 h after adult eclosion for females and adult males when larvae were fed only leaves from the top of a milkweed stem (dark) or leaves from the bottom of a milkweed stem (light). Different letters above bars within the same life stage indicate significant differences.

Leaf position significantly influenced performance across multiple developmental metrics (Table 2; Figure 1). Larvae fed top leaves were ~22% heavier on the sixth day of development, pupae were ~4% heavier, and female adults were ~10% heavier than those fed bottom leaves. Male adult mass was slightly higher for larvae fed top leaves, but this difference was not statistically significant. Finally, larvae fed top leaves pupated nearly one day earlier when fed top leaves than those fed bottom leaves.

### 3.3. Feeding Behavior Assays

In 24 h no-choice assays, neonates consumed similar amounts of leaf material when provided only top leaves (2.84 ± 0.28 mg) or bottom leaves (2.58 ± 0.31 mg) of milkweed plants, with no significant difference in biomass consumed (df = 1, 149; F = 0.270; *p* = 0.6042). However, neonates fed top leaves gained significantly more weight (5.02 ± 0.33 mg) than those fed bottom leaves (3.37 ± 0.24 mg; df = 1, 149; f = 13.366; *p* = 0.0004). Third instars provided with top leaves consumed significantly more biomass (19.35 ± 1.44 mg) than those provided bottom leaves (15.71 ± 0.84 mg; df = 1, 171; F = 9.154; *p* = 0.0029), and similarly gained more weight when fed top leaves (31.40 ± 2.10 mg) compared to bottom leaves (25.36 ± 1.64 mg; (df = 1, 171; F = 8.904; *p* = 0.0033).

**Figure 2:**
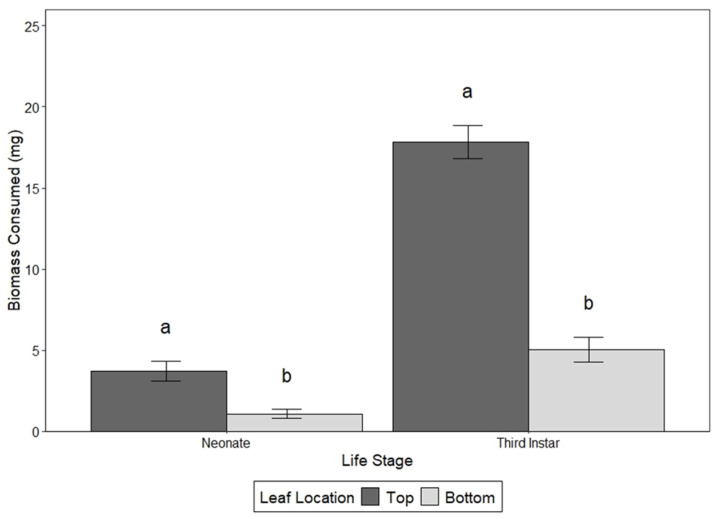
Bar graph with the average +/− standard deviation of the dry leaf biomass consumed of top leaves (dark) and bottom leaves (light) by neonates and third instars in the choice bioassays. Different letters above bars within the same life stage indicate significant differences.

In choice assays, both neonates and third instars showed a clear preference for younger vegetation (Figure 2). Neonates consumed significantly more leaf material from leaf disks taken from top leaves (2.02 ± 0.21 mg) than from leaf disks taken from bottom leaves (0.69 ± 0.11 mg; df = 1, 148; F = 20.942; *p* < 0.0001). Third instars also consumed substantially more top leaf material (17.83 ± 1.02 mg) than bottom leaf material (5.04 ± 0.75 mg; df = 1, 186; F = 104.033; *p* < 0.0001).

## 4. Discussion

Our results demonstrate differences in leaf characteristics between the top and the bottom of field-grown *Asclepias syriaca* stems. Top leaves were smaller in both length and width, required less force to puncture, and contained higher dry-weight percentages of total nitrogen. These findings are consistent with previous research showing that younger vegetative growth tends to be smaller, less tough, and more nutrient-rich than older leaves [20,22,23,24,25], and indicate that top milkweed leaves are both easier to consume and nutritionally valuable. As a result, top leaves may represent a more efficient and advantageous food source for developing monarch larvae. Although younger leaves have been reported to contain higher concentrations of defensive traits such as cardenolides and latex [13,21], monarch larvae are capable of tolerating these defenses due to their physiological adaptations for cardenolide sequestration [16] and behavioral adaptation of leaf trenching [11,48], respectively. Additionally, *A. syriaca* is considered an intermediately defended milkweed species in terms of cardenolide and latex levels, which has been correlated with optimal monarch survival across milkweed species [16,49,50]. This suggests that, although cardenolide and latex levels were not measured in this study, these defenses were likely within a tolerable range for monarch larvae, despite potential differences between leaf types. Overall, these findings support the hypotheses that milkweed quality varies seasonally and ontogenetically, and that younger milkweed vegetation offers higher quality resources than older growth.

Differences in leaf quality are known to influence lepidopteran growth and survival [34,35]. We found that monarch larvae fed exclusively on top leaves exhibited modest but consistent improvements in performance: greater mass at day six, heavier pupae, faster development, and heavier female adults compared to those fed bottom leaves. These traits are associated with increased adult fecundity [6,51] and may reduce vulnerability to natural enemies, such as predators and parasitoids, by shortening the duration of larval stages [52,53]. These effects, while modest in magnitude, suggest that access to younger leaves can enhance overall fitness in monarchs.

Behavioral assays reinforce these findings. Both neonates and third instars showed a clear preference for younger leaves when given a choice, indicating that larvae can distinguish leaf types and actively select higher quality tissue, as seen in other Lepidopteran species [54,55]. This behavioral preference provides potential justification for the observed oviposition bias toward young leaves, as female adults make food choices for their offspring [26,27,28,29,30,31], and why larvae are observed most frequently at the top of milkweed plants [9,33]. It also provides insight into natal ramet abandonment, a behavior that may occur when top leaves are depleted [9,41].

These ecological insights have direct implications for habitat management. Many studies have shown that strategically timed disturbance (e.g., mowing) of monarch habitat can stimulate milkweed regrowth, thereby increasing the availability of young, high-quality forage later in the season and supporting reproduction [29,30,31,32]. In addition to promoting reproduction, mowing may offer secondary benefits, such as reducing aphid infestations and creating enemy-free space for larvae [31,36]. However, benefits must be weighed against trade-offs. Milkweed senescence is a cue for the end of the breeding season and the onset of reproductive diapause and fall migration [56]. Promoting late-season regrowth risks disrupting phenological cues and migration timing, potentially creating conflict between extended breeding opportunities and migratory success [56]. Disturbance can also reduce floral resources that are preferred by adult monarchs [57] and utilized by other pollinators and herbivores [58], limit seed production, and cause direct mortality of eggs and larvae already present [29,30,31,32,36]. To balance these considerations, selective or rotational mowing over several years (i.e., leaving undisturbed areas to preserve phenological cues and floral availability) offers a more sustainable approach.

Beyond disturbance, other environmental factors likely shape milkweed quality and larval outcomes. Microhabitat light and shade can influence plant defenses and larval performance [59,60]. Soil fertility and broader environmental quality underpin the nutritional quality of host plants [61], which may in turn influence milkweed quality and monarch larval development. Drought-related stressors, including elevated temperatures and reduced water availability, can alter milkweed leaf traits, such as increased leaf nitrogen and decreased area and defensive traits [62] in ways that interact with monarch biology. Integrating these variables with milkweed phenology will be important for designing ecologically informed strategies that maintain high-quality host tissues throughout the breeding season.

Finally, while disturbance and favorable environmental conditions can increase egg recruitment, more eggs do not necessarily translate to more adult butterflies. Density-dependent processes, resource limitation, and natural enemy pressures can constrain cohort success [9,41]. Future research should evaluate how managed regrowth and complementary microhabitat features influence monarch fitness across landscapes and life stages, ensuring that these efforts deliver not just milkweed abundance, but milkweed quality at the right time.

Our findings underscore the importance of milkweed quality, life stage, and management in supporting monarch populations. Habitat interventions that align with monarch biology and seasonal dynamics, such as strategically timed mowing, may enhance reproductive success, but must be done with caution to avoid disturbing ecosystem function. Continued research should refine conservation practices to ensure restored habitats provide not only milkweed abundance, but milkweed quality at the right time, keeping strategies ecologically informed and regionally adaptive.

## Figures and Tables

**Table 1:** Leaf quality metrics.

Variable	Top Leaves Mean ± SD	Bottom Leaves Mean ± SD	F Statistic (df)	*p*-Value
Leaf length (cm)	10.5 ± 2.9	13.2 ± 5.1	24.444 (1, 214)	<0.0001
Leaf width (cm)	4.5 ± 1.2	7.2 ± 2.6	97.416 (1, 214)	<0.0001
Puncture force (lbs)	0.73 ± 0.022	0.89 ± 0.022	27.807 (1, 214)	<0.0001
Nitrogen content (% dry weight)	4.8 ± 0.18	3.1 ± 0.037	883.922 (1, 18)	<0.0001

**Table 2:** Monarch growth and development metrics.

Variable	Fed Top Leaves Mean ± SD	Fed Bottom Leaves Mean ± SD	F Statistic (df)	*p*-Value
Larval mass (Day 6, g)	0.12 ± 0.069	0.098 ± 0.066	5.219 (1, 264)	0.0231
Pupal mass (g)	1.10 ± 0.17	1.06 ± 0.15	4.734 (1, 254)	0.0305
Female adult mass (g)	0.44 ± 0.10	0.40 ± 0.06	6.967 (1, 99)	0.0096
Male adult mass (g)	0.47 ± 0.10	0.45 ± 0.08	1.595 (1, 88)	0.2100
Time to pupation (days)	14.06 ± 1.45	14.86 ± 1.79	15.610 (1, 254)	0.0001

## Data Availability

All data and associated files are available on GitHub: https://github.com/kelseyefisher/MilkweedLeafAgeMonarchSuccess.git. Accessed on 11 December 2025.

## References

[1] (2020). USFWS Monarch (*Danaus plexippus*) Species Status Assessment Report, Version 2.1. https://www.fws.gov/sites/default/files/documents/Monarch-Butterfly-SSA-Report-September-2020.pdf.

[2] USFWS Endangered and Threatened Wildlife and Plants; Threatened Species Status with Section 4(d) Rule for Monarch Butterfly and Designation of Critical Habitat. https://www.fws.gov/sites/default/files/documents/2024-12/threatened-species-status-with-section-4-d-rule-for-monarch-butterfly-and-designation-of-critical-habitat_0.pdf.

[3] Grant T.J., Fisher K.E., Krishnan N., Mullins A.N., Hellmich R.L., Sappington T.W., Adelman J.S., Coats J.R., Hartzler R.G., Pleasants J.M. (2022). Monarch butterfly ecology, behavior, and vulnerabilities in north central United States agricultural landscapes. BioScience.

[4] Zalucki M.P., Parry H.R., Zalucki J.M. (2016). Movement and egg laying in monarchs: To move or not to move, that is the equation. Austral Ecol..

[5] Blader T.R. (2018). Milkweed Patch Size Effects on Monarch Butterfly Oviposition Within Iowa Prairies and Roadsides. Master’s Thesis.

[6] Zalucki M.P., Kitching R.L. (1982). Dynamics of oviposition in *Danaus plexippus* (Insecta: Lepidoptera) on milkweed, *Asclepias* spp.. J. Zool..

[7] Zalucki M.P., Lammers J.H. (2010). Dispersal and egg shortfall in monarch butterflies: What happens when the matrix is cleaned up?. Ecol. Entomol..

[8] Grant T.J., Parry H.R., Zalucki M.P., Bradbury S.P. (2018). Predicting monarch butterfly (*Danaus plexippus*) movement and egg-laying with a spatially-explicit agent-based model: The role of monarch perceptual range and spatial memory. Ecol. Model..

[9] Fisher K.E., Hellmich R.L., Bradbury S.P. (2020). Estimates of common milkweed (*Asclepias syriaca*) utilization by monarch larvae (*Danaus plexippus*) and the significance of larval movement. J. Insect Conserv..

[10] Agrawal A.A., Fishbein M. (2006). Plant defense syndromes. Ecology.

[11] Zalucki M.P., Malcolm S.B. (1999). Plant latex and first instar monarch larval growth and survival on three North American milkweed species. J. Chem. Ecol..

[12] Zalucki M.P., Brower L.P., Alonso-M A. (2001). Detrimental effects of latex and cardiac glycosides on survival and growth of first-instar monarch butterfly larvae *Danaus plexippus* feeding on the sandhill milkweed *Asclepias humistrata*. Ecol. Entomol..

[13] Agrawal A.A., Konno K. (2009). Latex: A model for understanding mechanisms, ecology, and evolution of plant defense against herbivory. Annu. Rev. Ecol. Evol. Syst..

[14] Agrawal A.A., Hastings A.P., Patrick E.T., Knight A.C. (2014). Specificity of herbivore-induced hormonal signaling and defensive traits in five closely related milkweeds (*Asclepias* spp.). J. Chem. Ecol..

[15] Agrawal A.A. (2017). Hatching and defending. Monarchs and Milkweed: A Migrating Butterfly, a Poisonous Plant, and Their Remarkable Story of Coevolution.

[16] Petschenka G., Agrawal A.A. (2015). Milkweed butterfly resistance to plant toxins is linked to sequestration, not coping with a toxic diet. Proc. R. Soc. B Biol. Sci..

[17] Pocius V.M., Debinski D.M., Pleasants J.M., Bidne K.G., Hellmich R.L. (2018). Monarch butterflies do not place all of their eggs in one basket: Oviposition on nine Midwestern milkweed species. Ecosphere.

[18] Pocius V.M., Pleasants J.M., Debinski D.M., Bidne K.G., Hellmich R.L., Bradbury S.P., Blodgett S.L. (2018). Monarch butterflies show differential utilization of nine Midwestern milkweed species. Front. Ecol. Evol..

[19] Baker A.M., Redmond C.T., Malcolm S.B., Potter D.A. (2020). Suitability of native milkweed (*Asclepias*) species versus cultivars for supporting monarch butterflies and bees in urban gardens. PeerJ.

[20] Stamp N. (2003). Out of the quagmire of plant defense hypotheses. Q. Rev. Biol..

[21] Nelson C., Seiber J., Brower L. (1981). Seasonal and intraplant variation of cardenolide content in the California milkweed, *Asclepias eriocarpa*, and implications for plant defense. J. Chem. Ecol..

[22] Scriber J., Slansky F. (1981). The nutritional ecology of immature insects. Annu. Rev. Entomol..

[23] Thomas H., Stoddart J. (1990). Leaf senescence. Annu. Rev. Plant Biol..

[24] Slansky F., Stamp N., Casey T. (1993). Nutritional Ecology: The fundamental quest for nutrients. Caterpillars: Ecological and Envolutionary Constraints on Foraging.

[25] Lim P., Kim H., Nam H. (2007). Leaf senescence. Annu. Rev. Plant Biol..

[26] Borkin S.S. (1982). Notes on shifting distribution patterns and survival of immature *Danaus plexippus* (Lepidoptera: Danaidae) on the food plant *Asclepias syriaca*. Gt. Lakes Entomol..

[27] Urquhart F. (1987). The Monarch Butterfly: International Traveller.

[28] Bergström G., Rothschild M., Groth I., Crighton C. (1994). Oviposition by butterflies on young leaves: Investigation of leaf volatiles. Chemoecology.

[29] Fischer S.J., Williams E.H., Brower L.P., Palmiotto P.A. (2015). Enhancing monarch butterfly reproduction by mowing fields of common milkweed. Am. Midl. Nat..

[30] Alcock J., Brower L.P., Williams E.H. (2016). Monarch butterflies use regenerating milkweeds for reproduction in mowed hayfields in northern Virginia. J. Lepidopterists’ Soc..

[31] Haan N.L., Landis D.A. (2019). Grassland disturbance increases monarch butterfly oviposition and decreases arthropod predator abundance. Biol. Conserv..

[32] Knight S.M., Norris D.R., Derbyshire R., Flockhart D.T.T. (2019). Strategic mowing of roadside milkweeds increases monarch butterfly oviposition. Glob. Ecol. Conserv..

[33] Lizotte-Hall S.E., Hartzler R.G. (2019). Effect of postemergence fomesafen application on common milkweed (*Asclepias syriaca*) growth and utilization by monarchs (*Danaus plexippus*). Crop Prot..

[34] Stamp N.E., Bowers M.D. (1990). Phenology of nutritional differences between new and mature leaves and its effect on caterpillar growth. Ecol. Entomol..

[35] Jordano D., Gomariz G. (1994). Variation in phenology and nutritional quality between host plants and its effect on larval performance in a specialist butterfly, *Zerynthia rumina*. Entomol. Exp. Appl..

[36] Haan N.L., Landis D.A. (2019). The importance of shifting disturbance regimes in monarch butterfly decline and recovery. Front. Ecol. Evol..

[37] Urquhart F. (1960). The Monarch Butterfly.

[38] Rawlins J.E., Lederhouse R.C. (1981). Developmental influences of thermal behavior on monarch caterpillars (*Danaus plexippus*): An adaptation for migration (Lepidoptera: Nymphalidae: Danainae). J. Kans. Entomol. Soc..

[39] Zalucki M.P., Rochester W., Oberhauser K.S., Solensky M.J. (2004). Spatial and temporal population dynamics of monarchs down-under: Lessons for North America. The Monarch Butterfly: Biology and Conservation.

[40] De Anda A., Oberhauser K.S., Oberhauser K.S., Nail K.R., Altizer S. (2015). Invertebrate natural enemies and stage-specific mortality rates of monarch eggs and larvae. Monarchs in a Changing World.

[41] Fisher K.E., Bradbury S.P. (2022). Plant abandonment behavior and fitness of monarch larvae (*Danaus plexippus*) is not influenced by an intraspecific competitor. J. Insect Conserv..

[42] Halsch C.A., Code A., Hoyle S.M., Fordyce J.A., Baert N., Forister M.L. (2020). Pesticide contamination of milkweeds across the agricultural, urban, and open spaces of low-elevation northern California. Front. Ecol. Evol..

[43] Fisher K.E., Mason C.E., Flexner J.L., Hough-Goldstein J., McDonald J.H. (2017). Survivorship of z-pheromone race European corn borer (Lepidoptera: Crambidae) on a range of host plants varying in defensive chemistry. J. Econ. Entomol..

[44] Fisher K.E., Flexner J.L., Mason C.E. (2020). Plant preferences of z-pheromone race *Ostrinia nubilalis* (Lepidoptera: Crambidae) based on leaf tissue consumption rates. J. Econ. Entomol..

[45] Rasband W.S. ImageJ, National Institute of Health, Bethesda, Maryland, USA. https://imagej.net/ij/.

[46] RStudio Team RStudio: Integrated Development for R 2025. https://rstudio.com/.

[47] Lenth R., Singmann H., Love J., Buerkner P., Herve M. Emmeans: Estimated Marginal Means, Aka Least-Squares Means 2020. https://CRAN.R-project.org/package=emmeans.

[48] Zalucki M.P., Brower L.P. (1992). Survival of first instar larvae of *Danaus plexippus* (Lepidoptera: Danainae) in relation to cardiac glycoside and latex content of *Asclepias humistrata* (Asclepiadaceae). Chemoecology.

[49] Agrawal A.A., Fishbein M., Jetter R., Salminen J., Goldstein J.B., Freitag A.E., Sparks J.P. (2009). Phylogenetic ecology of leaf surface traits in the milkweeds (*Asclepias* spp.): Chemistry, ecophysiology, and insect behavior. New Phytol..

[50] Rasmann S., Agrawal A.A. (2011). Evolution of specialization: A phylogenetic study of host range in the red milkweed beetle (*Tetraopes tetraophthalmus*). Am. Nat..

[51] Oberhauser K.S. (1997). Fecundity, Lifespan and egg mass in butterflies: Effects of male-derived nutrients and female size. Funct. Ecol..

[52] Benrey B., Dennoi R.F. (1997). The slow-growth-high-mortality hypothesis: A test using the cabbage butterfly. Ecology.

[53] Chen K., Chen Y. (2018). Slow-growth high-mortality: A meta-analysis for insects. Insect Sci..

[54] Bernays E., Chapman R. (1994). Host-Plant Selection by Phytophagous Insects.

[55] Singer M.C. (1971). Evolution of food-plant preference in the butterfly *Euphydryas editha*. Evolution.

[56] Majewska A.A., Altizer S. (2019). Exposure to non-native tropical milkweed promotes reproductive development in migratory monarch butterflies. Insects.

[57] Fisher K.E., Snyder B.R., Bradbury S.P. (2023). Blooming forbs utilized by breeding-season *Danaus plexippus* in the USA north-central region. J. Lepitopterists’ Soc..

[58] Morse D.H. (1985). Milkweeds and their visitors. Sci. Am..

[59] Agrawal A.A., Kearney E.E., Hastings A.P., Ramsey T.E. (2012). Attenuation of the jasmonate burst, plant defensive traits, and resistance to specialist monarch caterpillars on shaded common milkweed (*Asclepias syriaca*). J. Chem. Ecol..

[60] Boone M., McKnight S., King K., Henry E., Schultz C. (2025). Late summer western monarch survival is affected by shade environment and milkweed species 2025. J. Insect Conserv..

[61] Aytenew M., Bore G. (2020). Effects of organic amendments on soil fertility and environmental quality: A review. J. Plant Sci..

[62] Couture J., Serbin S., Townsend P. (2015). Elevated temperature and periodic water stress alter growth and quality of common milkweed (*Asclepias syriaca*) and monarch (*Danaus plexippus*) larval performance. Arthropod-Plant Interact..

